# 2-Cyano­quinolin-1-ium nitrate

**DOI:** 10.1107/S1600536810039243

**Published:** 2010-10-09

**Authors:** Wan-Sin Loh, Madhukar Hemamalini, Hoong-Kun Fun

**Affiliations:** a X-ray Crystallography Unit, School of Physics, Universiti Sains Malaysia, 11800 USM, Penang, Malaysia

## Abstract

A proton is transferred from the nitric acid to the N atom of 2-cyano­quinoline during crystallization, resulting in the formation of the title salt, C_10_H_7_N_2_^+^·NO_3_^−^. The quinolinium ring system is approximately planar, with a maximum deviation of 0.013 (3) Å. In the crystal, a very asymmetric bifurcated N—H⋯(O,O) hydrogen bond to two O atoms of an adjacent nitrate anion occurs, generating an *R*_2_^1^(4) ring motif. C—H⋯O hydrogen bonds link the ions into sheets stacking along the *a* axis.

## Related literature

For background to and the biological activities of quinoline derivatives, see: Loh, Quah *et al.* (2010*a* ▶,*b* ▶); Loh *et al.* (2010 ▶); Sasaki *et al.* (1998 ▶); Reux *et al.* (2009 ▶); Morimoto *et al.* (1991 ▶); Michael (1997 ▶); Markees *et al.* (1970 ▶); Campbell *et al.* (1988 ▶). For the hydrogen-bond motif, see: Bernstein *et al.* (1995 ▶). For related structures, see: Loh, Quah *et al.* (2010*a* ▶,*b* ▶); Loh *et al.* (2010 ▶). For the stability of the temperature controller used for the data collection, see: Cosier & Glazer (1986 ▶). For bond-length data, see: Allen *et al.* (1987 ▶).
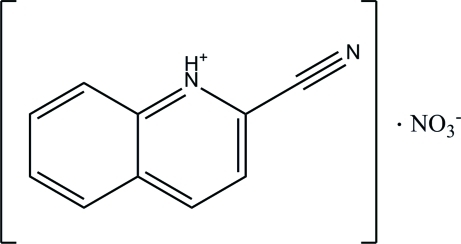


## Experimental

### 

#### Crystal data

C_10_H_7_N_2_^+^·NO_3_^−^
*M* *_r_* = 217.19
Monoclinic, 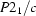

*a* = 3.6969 (1) Å
*b* = 17.7031 (3) Å
*c* = 14.6029 (2) Å
β = 95.802 (1)°
*V* = 950.81 (3) Å^3^
*Z* = 4
Mo *K*α radiation
μ = 0.12 mm^−1^
*T* = 100 K
0.44 × 0.18 × 0.07 mm

#### Data collection

Bruker SMART APEXII CCD diffractometer
Absorption correction: multi-scan (*SADABS*; Bruker, 2009 ▶) *T*_min_ = 0.951, *T*_max_ = 0.992
16184 measured reflections
2758 independent reflections
2103 reflections with *I* > 2σ(*I*)
*R*_int_ = 0.041

#### Refinement

*R*[*F* ^2^ > 2σ(*F* ^2^)] = 0.045
*wR*(*F* ^2^) = 0.122
*S* = 1.05
2758 reflections
170 parameters
H atoms treated by a mixture of independent and constrained refinement
Δρ_max_ = 0.37 e Å^−3^
Δρ_min_ = −0.29 e Å^−3^

### 

Data collection: *APEX2* (Bruker, 2009 ▶); cell refinement: *SAINT* (Bruker, 2009 ▶); data reduction: *SAINT*; program(s) used to solve structure: *SHELXTL* (Sheldrick, 2008 ▶); program(s) used to refine structure: *SHELXTL*; molecular graphics: *SHELXTL*; software used to prepare material for publication: *SHELXTL* and *PLATON* (Spek, 2009 ▶).

## Supplementary Material

Crystal structure: contains datablocks global, I. DOI: 10.1107/S1600536810039243/hb5669sup1.cif

Structure factors: contains datablocks I. DOI: 10.1107/S1600536810039243/hb5669Isup2.hkl

Additional supplementary materials:  crystallographic information; 3D view; checkCIF report

## Figures and Tables

**Table 1:** Hydrogen-bond geometry (Å, °)

*D*—H⋯*A*	*D*—H	H⋯*A*	*D*⋯*A*	*D*—H⋯*A*
N1—H1*N*1⋯O1	0.95	2.56	3.2376 (15)	128
N1—H1*N*1⋯O3	0.95	1.60	2.5432 (14)	172
C5—H5*A*⋯O3^i^	0.943 (16)	2.497 (16)	3.2835 (16)	141.0 (15)
C7—H7*A*⋯O2^ii^	0.976 (16)	2.473 (16)	3.3641 (16)	151.8 (12)
C8—H8*A*⋯O2^iii^	0.977 (18)	2.391 (19)	3.3355 (17)	162.4 (15)

Symmetry codes: (i) 
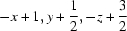
; (ii) 
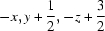
; (iii) 
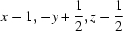
.

**Fig. 1.**
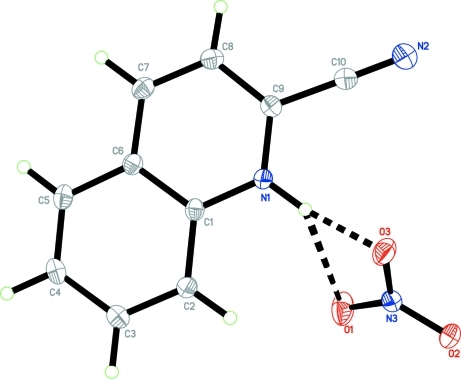
The molecular structure of the title compound showing 50% probability displacement ellipsoids for non-H atoms. The R21(4) ring motif which indicates the bifurcated hydrogen bond is shown.

**Fig. 2.**
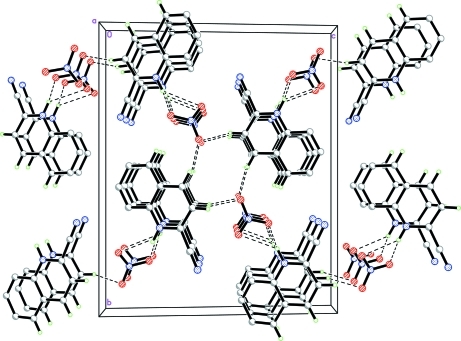
The crystal structure of the title compound, viewed along the a axis.

## supplementary crystallographic information

### Comment

Recently, hydrogen-bonding patterns involving quinoline and its derivatives with organic acid have been investigated (Loh *at al.*, 2010*a,b*; Loh *et al.*,  2010). Syntheses of the quinoline derivatives were discussed earlier (Sasaki *et al.*, 1998; Reux *et al.*, 2009). Quinolines and their derivatives are very important compounds because of their wide occurrence in natural products (Morimoto *et al.*, 1991; Michael, 1997) and biologically active compounds (Markees *et al.*, 1970; Campbell *et al.*, 1988). Heterocyclic molecules containing cyano group are useful as drug intermediates. Herein we report the synthesis of 2-cyanoquinolin-1-ium nitrate.

The asymmetric unit of the title compound (Fig. 1) consists of one 2-cyanoquinolin-1-ium cation (C1–C10/N1/N2) and one nitrate anion (N3/O1–O3). One proton is transferred from the hydroxyl group of nitrate to the atom N1 of 2-cyanoquinoline during the crystallization, resulting in the formation of salt. The quinoline ring system (C1–C9/N1) is approximately planar with a maximum deviation of 0.013 (3) Å at atom C6. The *R*_2_^1^(4) ring motif (Fig. 1; Bernstein *et al.*, 1995) indicates a bifurcated hydrogen bond from N1–H1N1 to the two acceptors (O1/O3). Bond lengths (Allen *et al.*, 1987) and angles are within the normal ranges and are comparable to the related structures (Loh *et al.*, 2010*a,b*; Loh *et al.*,  2010).

In the crystal packing (Fig. 2), intermolecular N1—H1N1···O1, N1—H1N1···O3, C5—H5A···O3, C7—H7A···O2 and C8—H8A···O2 hydrogen bonds (Table 1) link the molecules into two-dimensional planes stacking along the *a* axis.

### Experimental

A few drops of nitric acid were added to a hot methanol solution (20 ml) of quinoline-2-carbonitrile (39 mg, Aldrich) which had been warmed over a magnetic stirrer hotplate for a few minutes. The resulting solution was allowed to cool slowly to room temperature. Colourless plates of (I) appeared after a few days.

### Refinement

All H atoms were located from a difference Fourier map. H1N1 was fixed at its found position with bond length of N—H being 0.9481 Å. The remaining H atoms were refined freely with the bond lengths of C—H being 0.943 (17) to 0.998 (17) Å.

### Crystal data

**Table:**

C_10_H_7_N_2_^+^·NO_3_^−^	*F*(000) = 448
*M**_r_* = 217.19	*D*_x_ = 1.517 Mg m^−^^3^
Monoclinic, *P*2_1_/*c*	Mo *K*α radiation, λ = 0.71073 Å
Hall symbol: -P 2ybc	Cell parameters from 3861 reflections
*a* = 3.6969 (1) Å	θ = 2.7–31.0°
*b* = 17.7031 (3) Å	µ = 0.12 mm^−^^1^
*c* = 14.6029 (2) Å	*T* = 100 K
β = 95.802 (1)°	Plate, colourless
*V* = 950.81 (3) Å^3^	0.44 × 0.18 × 0.07 mm
*Z* = 4	

### Data collection

**Table:**

Bruker SMART APEXII CCD diffractometer	2758 independent reflections
Radiation source: fine-focus sealed tube	2103 reflections with *I* > 2σ(*I*)
graphite	*R*_int_ = 0.041
φ and ω scans	θ_max_ = 30.0°, θ_min_ = 1.8°
Absorption correction: multi-scan (*SADABS*; Bruker, 2009)	*h* = −5→5
*T*_min_ = 0.951, *T*_max_ = 0.992	*k* = −24→24
16184 measured reflections	*l* = −20→20

### Refinement

**Table:**

Refinement on *F*^2^	Primary atom site location: structure-invariant direct methods
Least-squares matrix: full	Secondary atom site location: difference Fourier map
*R*[*F*^2^ > 2σ(*F*^2^)] = 0.045	Hydrogen site location: inferred from neighbouring sites
*wR*(*F*^2^) = 0.122	H atoms treated by a mixture of independent and constrained refinement
*S* = 1.05	*w* = 1/[σ^2^(*F*_o_^2^) + (0.0614*P*)^2^ + 0.2227*P*] where *P* = (*F*_o_^2^ + 2*F*_c_^2^)/3
2758 reflections	(Δ/σ)_max_ < 0.001
170 parameters	Δρ_max_ = 0.37 e Å^−^^3^
0 restraints	Δρ_min_ = −0.29 e Å^−^^3^

### Special details

**Table:**

Experimental. The crystal was placed in the cold stream of an Oxford Cryosystems Cobra open-flow nitrogen cryostat (Cosier & Glazer, 1986) operating at 100.0 (1) K.
Geometry. All e.s.d.'s (except the e.s.d. in the dihedral angle between two l.s. planes) are estimated using the full covariance matrix. The cell e.s.d.'s are taken into account individually in the estimation of e.s.d.'s in distances, angles and torsion angles; correlations between e.s.d.'s in cell parameters are only used when they are defined by crystal symmetry. An approximate (isotropic) treatment of cell e.s.d.'s is used for estimating e.s.d.'s involving l.s. planes.
Refinement. Refinement of *F*^2^ against ALL reflections. The weighted *R*-factor *wR* and goodness of fit *S* are based on *F*^2^, conventional *R*-factors *R* are based on *F*, with *F* set to zero for negative *F*^2^. The threshold expression of *F*^2^ > σ(*F*^2^) is used only for calculating *R*-factors(gt) *etc*. and is not relevant to the choice of reflections for refinement. *R*-factors based on *F*^2^ are statistically about twice as large as those based on *F*, and *R*- factors based on ALL data will be even larger.

### Fractional atomic coordinates and isotropic or equivalent isotropic displacement parameters (Å^2^)

**Table:**

	*x*	*y*	*z*	*U*_iso_*/*U*_eq_	
O1	0.1851 (3)	0.21342 (6)	0.92466 (7)	0.0314 (3)	
O2	0.4106 (3)	0.10064 (5)	0.91484 (7)	0.0255 (3)	
O3	0.4296 (3)	0.17922 (5)	0.80150 (6)	0.0258 (3)	
N1	0.2524 (3)	0.30942 (6)	0.73844 (7)	0.0144 (2)	
H1N1	0.3241	0.2630	0.7672	0.079 (8)*	
N2	−0.1335 (4)	0.17939 (7)	0.58610 (8)	0.0265 (3)	
N3	0.3373 (3)	0.16413 (6)	0.88321 (7)	0.0196 (3)	
C1	0.3582 (3)	0.37566 (7)	0.78076 (8)	0.0142 (3)	
C2	0.5593 (4)	0.37429 (7)	0.86850 (8)	0.0161 (3)	
C3	0.6627 (4)	0.44133 (7)	0.90990 (9)	0.0176 (3)	
C4	0.5697 (4)	0.51140 (7)	0.86660 (9)	0.0181 (3)	
C5	0.3739 (4)	0.51378 (7)	0.78226 (9)	0.0175 (3)	
C6	0.2637 (3)	0.44559 (7)	0.73637 (8)	0.0148 (3)	
C7	0.0675 (4)	0.44419 (7)	0.64838 (9)	0.0176 (3)	
C8	−0.0316 (4)	0.37627 (7)	0.60703 (9)	0.0175 (3)	
C9	0.0658 (4)	0.30972 (7)	0.65509 (8)	0.0158 (3)	
C10	−0.0406 (4)	0.23670 (7)	0.61661 (9)	0.0184 (3)	
H2A	0.619 (4)	0.3267 (9)	0.8975 (11)	0.020 (4)*	
H3A	0.805 (5)	0.4406 (9)	0.9717 (12)	0.023 (4)*	
H4A	0.649 (5)	0.5595 (10)	0.8975 (11)	0.028 (4)*	
H5A	0.304 (5)	0.5598 (9)	0.7531 (11)	0.024 (4)*	
H7A	0.002 (4)	0.4918 (9)	0.6174 (11)	0.020 (4)*	
H8A	−0.166 (5)	0.3735 (10)	0.5461 (13)	0.030 (4)*	

### Atomic displacement parameters (Å^2^)

**Table:**

	*U*^11^	*U*^22^	*U*^33^	*U*^12^	*U*^13^	*U*^23^
O1	0.0486 (7)	0.0188 (5)	0.0295 (6)	0.0018 (5)	0.0167 (5)	−0.0018 (4)
O2	0.0375 (6)	0.0142 (5)	0.0234 (5)	−0.0007 (4)	−0.0040 (4)	0.0047 (4)
O3	0.0438 (7)	0.0173 (5)	0.0170 (5)	0.0075 (4)	0.0072 (4)	0.0026 (4)
N1	0.0169 (6)	0.0120 (5)	0.0142 (5)	0.0006 (4)	0.0010 (4)	0.0000 (4)
N2	0.0331 (7)	0.0217 (6)	0.0237 (6)	−0.0029 (5)	−0.0020 (5)	−0.0014 (5)
N3	0.0255 (6)	0.0153 (5)	0.0172 (5)	−0.0027 (4)	−0.0015 (4)	−0.0003 (4)
C1	0.0146 (6)	0.0126 (6)	0.0159 (6)	0.0002 (4)	0.0035 (5)	−0.0005 (4)
C2	0.0180 (6)	0.0145 (6)	0.0156 (6)	0.0004 (5)	0.0013 (5)	0.0005 (4)
C3	0.0164 (6)	0.0187 (6)	0.0177 (6)	−0.0017 (5)	0.0020 (5)	−0.0024 (5)
C4	0.0180 (7)	0.0151 (6)	0.0216 (6)	−0.0035 (5)	0.0044 (5)	−0.0037 (5)
C5	0.0200 (7)	0.0128 (6)	0.0204 (6)	0.0002 (5)	0.0053 (5)	0.0002 (5)
C6	0.0146 (6)	0.0140 (6)	0.0160 (6)	0.0009 (4)	0.0027 (5)	0.0008 (4)
C7	0.0185 (7)	0.0166 (6)	0.0177 (6)	0.0030 (5)	0.0023 (5)	0.0023 (5)
C8	0.0179 (6)	0.0192 (6)	0.0153 (6)	0.0016 (5)	0.0008 (5)	0.0009 (5)
C9	0.0157 (6)	0.0159 (6)	0.0158 (6)	−0.0003 (5)	0.0017 (5)	−0.0016 (4)
C10	0.0190 (7)	0.0192 (6)	0.0166 (6)	0.0004 (5)	0.0003 (5)	0.0005 (5)

### Geometric parameters (Å, °)

**Table:**

O1—N3	1.2301 (15)	C3—H3A	0.998 (17)
O2—N3	1.2347 (14)	C4—C5	1.3646 (19)
O3—N3	1.3015 (14)	C4—H4A	0.993 (18)
N1—C9	1.3370 (16)	C5—C6	1.4203 (17)
N1—C1	1.3642 (15)	C5—H5A	0.943 (17)
N1—H1N1	0.9481	C6—C7	1.4104 (18)
N2—C10	1.1466 (18)	C7—C8	1.3784 (18)
C1—C2	1.4152 (17)	C7—H7A	0.976 (17)
C1—C6	1.4243 (16)	C8—C9	1.3998 (18)
C2—C3	1.3686 (18)	C8—H8A	0.977 (18)
C2—H2A	0.959 (16)	C9—C10	1.4480 (18)
C3—C4	1.4188 (18)		
			
C9—N1—C1	120.45 (10)	C3—C4—H4A	119.9 (10)
C9—N1—H1N1	120.2	C4—C5—C6	120.01 (12)
C1—N1—H1N1	119.3	C4—C5—H5A	122.1 (10)
O1—N3—O2	123.76 (12)	C6—C5—H5A	117.9 (10)
O1—N3—O3	118.74 (11)	C7—C6—C5	122.77 (11)
O2—N3—O3	117.50 (11)	C7—C6—C1	118.63 (11)
N1—C1—C2	119.72 (11)	C5—C6—C1	118.60 (12)
N1—C1—C6	119.68 (11)	C8—C7—C6	120.25 (12)
C2—C1—C6	120.60 (11)	C8—C7—H7A	120.5 (10)
C3—C2—C1	118.87 (12)	C6—C7—H7A	119.2 (10)
C3—C2—H2A	121.7 (10)	C7—C8—C9	118.10 (12)
C1—C2—H2A	119.4 (10)	C7—C8—H8A	122.1 (10)
C2—C3—C4	121.13 (12)	C9—C8—H8A	119.8 (10)
C2—C3—H3A	119.1 (9)	N1—C9—C8	122.87 (11)
C4—C3—H3A	119.8 (9)	N1—C9—C10	116.40 (11)
C5—C4—C3	120.79 (12)	C8—C9—C10	120.72 (12)
C5—C4—H4A	119.3 (10)	N2—C10—C9	178.33 (15)
			
C9—N1—C1—C2	−179.23 (11)	C2—C1—C6—C7	179.11 (11)
C9—N1—C1—C6	1.04 (18)	N1—C1—C6—C5	179.37 (11)
N1—C1—C2—C3	−179.94 (12)	C2—C1—C6—C5	−0.36 (18)
C6—C1—C2—C3	−0.21 (19)	C5—C6—C7—C8	179.86 (12)
C1—C2—C3—C4	0.39 (19)	C1—C6—C7—C8	0.40 (19)
C2—C3—C4—C5	0.0 (2)	C6—C7—C8—C9	0.44 (19)
C3—C4—C5—C6	−0.6 (2)	C1—N1—C9—C8	−0.16 (19)
C4—C5—C6—C7	−178.69 (12)	C1—N1—C9—C10	−178.81 (11)
C4—C5—C6—C1	0.76 (19)	C7—C8—C9—N1	−0.6 (2)
N1—C1—C6—C7	−1.15 (18)	C7—C8—C9—C10	177.99 (12)

### Hydrogen-bond geometry (Å, °)

**Table:**

*D*—H···*A*	*D*—H	H···*A*	*D*···*A*	*D*—H···*A*
N1—H1N1···O1	0.95	2.56	3.2376 (15)	128
N1—H1N1···O3	0.95	1.60	2.5432 (14)	172
C5—H5A···O3^i^	0.943 (16)	2.497 (16)	3.2835 (16)	141.0 (15)
C7—H7A···O2^ii^	0.976 (16)	2.473 (16)	3.3641 (16)	151.8 (12)
C8—H8A···O2^iii^	0.977 (18)	2.391 (19)	3.3355 (17)	162.4 (15)

Symmetry codes: (i) −*x*+1, *y*+1/2, −*z*+3/2; (ii) −*x*, *y*+1/2, −*z*+3/2; (iii) *x*−1, −*y*+1/2, *z*−1/2.
